# Enhancing colorectal cancer screening in high‐risk population through fecal immunochemical test surveillance: Results from a surveillance program

**DOI:** 10.1002/cam4.70145

**Published:** 2024-10-20

**Authors:** Hai Qin, Mingqing Zhang, Guanglu Zhang, Lizhong Zhao, Huan Zhang, Weituo Zhang, Yijia Wang, Xipeng Zhang, Li Xie, Biyun Qian

**Affiliations:** ^1^ Department of Colorectal Surgery, Tianjin Union Medical Center Nankai University Tianjin China; ^2^ Department of Preventive and health care, Tianjin Union Medical Center Nankai University Tianjin China; ^3^ Colorectal Cancer Screening Office Tianjin Institute of Coloproctology Tianjin China; ^4^ Hongqiao International Institute of Medicine, Shanghai Tongren Hospital and School of Public Health Shanghai Jiao Tong University School of Medicine Shanghai China; ^5^ Cancer Prevention Center Tianjin Medical University Cancer Institute and Hospital, National Clinical Research Center for Cancer Tianjin China; ^6^ Clinical Research Institute, Shanghai Jiao Tong University School of Medicine Shanghai China; ^7^ Department of Pathology, Tianjin Union Medical Center Nankai University Tianjin China; ^8^ Laboratory of Oncologic Molecular Medicine, Tianjin Union Medical Center Nankai University Tianjin China; ^9^ Shanghai Clinical Research Promotion and Development Center Shanghai Hospital Development Center Shanghai China

**Keywords:** cancer screening, colorectal cancer, fecal immunochemical testing, surveillance

## Abstract

**Background:**

Current guidelines recommend colonoscopy‐based surveillance to decrease the risk of colorectal cancer (CRC) among these participants with above‐average risk. The fecal immunochemical test (FIT) holds promise as a viable alternative surveillance tool, but the existing evidence regarding the use of settings remains limited. Therefore, our aim is to evaluate the CRC incidence rates in individuals with above‐average CRC risk and the relationship between FIT surveillance and CRC incidence.

**Methods:**

The retrospective cohort study was performed based on the CRC screening program between January 2012 and December 2022, in Tianjin, China. This cohort study included 12,515 participants aged 40–74 years with above‐average risk. The primary outcomes were the incidence rates of CRC and advanced colorectal neoplasia which were expressed as the number of events per 100,000 person‐years. Hazard ratios (HRs) and 95% confidence intervals (CIs) were calculated using Cox proportional hazards models.

**Results:**

We included 12,515 participants aged 40–74 years, of whom 4980 received subsequent FIT surveillance during the study period. Among these participants, 51 CRC cases occurred in the non‐FIT surveillance group (incidence rate, 233.88 per 100,000 person‐years) and there were 29 cases of CRC in the FIT surveillance group (incidence rate, 184.85 per 100,000 person‐years), resulting in an incidence rate ratio (IRR) of 0.58 (95% CI, 0.37–0.91). Meanwhile, 428 advanced colorectal neoplasia cases were reported in the non‐FIT surveillance group, while 269 cases occurred in the FIT surveillance group, with significantly lower incidence of advanced colorectal neoplasia in the FIT surveillance group (IRR: 0.64; 95% CI, 0.55–0.74). Compared with the non‐FIT surveillance group, the FIT surveillance group had a 54% decreased risk of developing CRC (HR, 0.46; 95% CI, 0.29–0.74) and a 45% decreased risk of developing advanced colorectal neoplasia (HR, 0.55; 95% CI, 0.47–0.64).

**Conclusions:**

In this retrospective cohort study, above‐average risk individuals who received subsequent FIT in the intervals between colonoscopies were associated with a reduction of CRC and advanced colorectal neoplasia incidence, which indicated the value and utility of FIT in the surveillance program.

## Graphical abstract

Colorectal cancer remains a major public health concern and there is a continuing need to refine and develop effective strategies for early detection and prevention, particularly in populations at higher than average risk. Our study investigates the effectiveness and benefits of Fecal Immunochemical Test (FIT) surveillance in individuals with high‐risk for colorectal cancer. Key findings from our study indicate that FIT surveillance in high‐risk population demonstrates promising results in terms of early detection and prevention of colorectal cancer and advanced colorectal neoplasia.

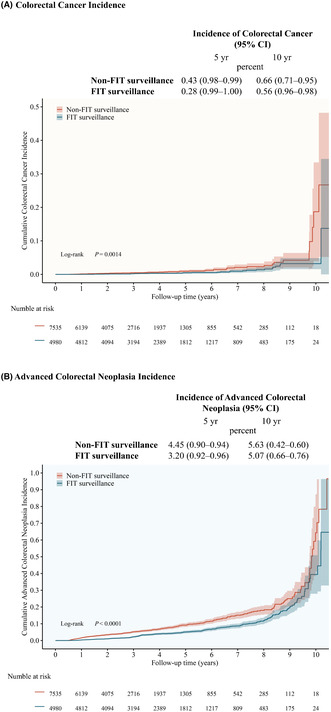

## 1 INTRODUCTION

Colorectal cancer (CRC) is the second most common cancer in China, and the incidence rate of CRC has increased rapidly in recent years.^1^ There is a continuing need to refine and develop effective strategies for early detection and prevention, particularly in populations at higher than average risk. Current guidelines recommend colonoscopy‐based surveillance to decrease the risk of CRC among these participants with previously detected adenomatous polyps.^2^, ^3^, ^4^ Surveillance combined with clinical findings at the time of colonoscopy recommends follow‐up colonoscopy within 3 years after removal of above‐average risk adenomas and within 5 years for low‐risk adenoma participants, which may be extended up to 7–10 years in the average‐risk population.^2^, ^5^, ^6^, ^7^, ^8^, ^9^ However, there is an urgent and inevitable concern is this surveillance schedule may lead to significantly increased demand for endoscopic examination, potentially exceeding the colonoscopy capacity, especially in low resource regions.

Several factors contribute to this situation. The widespread implementation of annual colorectal cancer screening programs is influenced by factors such as population growth among aging individuals and changes in lifestyles enhancing CRC screening rates.^10^ Moreover, individuals with increased awareness of colorectal cancer screening and prevention, along with active promotion and publicity by governments, healthcare institutions, and relevant organizations, have positioned colonoscopy examinations as the primary method for CRC screening.^11^, ^12^ As a result, more individuals are encouraged to seek screening services and undergo colonoscopy examinations. Regular surveillance after screening can help doctors assess the colon health of individuals and intervene in a timely manner to prevent the potential development of colorectal cancer.^2^, ^5^ Therefore, participants need to undergo regular colonoscopy examinations after screening to ensure timely detection and management of any abnormalities, which increased demand means that healthcare facilities and medical professionals need to provide more colonoscopy examination resources ensure that patients receive appropriate monitoring and treatment. Whereas resource limitations, including inadequate endoscopic equipment, manpower, and medical resources limit the capacity of colonoscopy and pose challenges in meeting the growing demand for colonoscopy surveillance. Such an escalation in demand for endoscopies was recently observed in Netherlands with a spike in the rates of primary care referrals for suspected CRC due to the widespread implementation of CRC screening.^13^

In addition to colonoscopy, fecal immunochemical testing (FIT) has been widely adopted in screening programs for CRC worldwide among participants with unknown risk^14^, ^15^ but has been largely overlooked in clinical studies as a method for surveillance among participants with elevated risk. Some recent studies reported that FIT testing could potentially serve as a viable alternative strategy for surveillance in participants with negative colonoscopy results. A study of population‐based screening programs in Taiwan found that FIT testing after a negative colonoscopy may reduce the risk of developing CRC.^16^ Another study in the United Kingdom found that using annual FIT instead of colonoscopy every 3 years could reduce colonoscopy procedures by 71% among intermediate‐risk participants (three to four small adenomas or one ≥10 mm), significantly lowering costs.^17^ However, data from other regions is still lacking, and furthermore, whether individuals with no neoplasia or non‐advanced neoplasia based on primary colonoscopy should routinely undergo FIT screening remains controversial.

In Asia and other resource‐limited regions, colonoscopy‐based surveillance can potentially greatly increase clinical workload, in addition to requiring staff with advanced training, and imposing financial burdens.^2^ Given these limitations, FIT presents an attractive alternative to CRC monitoring by colonoscopy. However, before adopting FIT for surveillance, more research is necessary to determine its performance in monitoring CRC in above‐average risk populations. Currently, one CRC screening program in Tianjin differentiates it from the rest of China in that above‐average risk individuals were invited to participate a follow‐up CRC surveillance cohort. To our knowledge, no studies in a Chinese population have yet investigated the effectiveness of FIT surveillance among participant with no neoplasia, non‐advanced colorectal neoplasia, or advanced colorectal neoplasia. Our current study, therefore, aims to investigate the performance of FIT in preventing advanced colorectal neoplasia and CRC in individuals with above‐average risk.

## 2 METHODS

### 2.1 Study setting and population

This retrospective cohort study included individuals enrolled in the long‐term CRC surveillance program in Tianjin between January 2012 and December 2022. Enrolled individuals were eligible if with above‐average risk for colorectal neoplasia or cancer due to: (1) a prior history of polyps, (2) or a family history of CRC, gastrointestinal symptoms, (3) or a previous positive FIT result. The exclusion criteria were as follows: (1) participants with invalid FIT results, (2) participants with follow‐up time <6 months due to detection of CRC or whatever reason for rapid follow up, (3) age <40 years or age >74 years, (4) missing covariates and inadequate bowel cleansing. The study population was recruited from 16 districts in Tianjin, China. All participants provided written informed consent. The study was approved by the ethics committees of the Tianjin Health Commission (ethical approval number 2023C04).

Community health service centers organize the screening participants to fill out informed consent forms, ensuring that they are fully aware and informed. They will conduct high risk factor questionnaires and FIT for colorectal cancer screening. Patients were assessed to the positive HRFQ due to: (1) a prior history of polyps, (2) a family history of CRC, (3) two or more symptoms of the following: a history of chronic constipation, a history of chronic diarrhea, a history of bloody mucous stools, adverse life events (e.g., divorce, death of a close relative, etc.), a history of chronic appendicitis or appendectomy, a history of chronic cholecystitis or gallstones. Participants who have positive results on either the questionnaire or the FIT are identified as high‐risk individuals, recommended to undergo a free colonoscopy.

### 2.2 Surveillance procedures

Follow‐up visits are conducted for high‐risk individuals identified previously. The district health commission organizes community health service centers to carry out the follow‐up work. High‐risk Individuals with advanced adenoma, and non‐advanced adenoma were provided regular colonoscopy health tips. It is recommended that all high‐risk individuals undergo annual FIT at community health service centers, with dedicated investigators providing telephone reminders.

Individuals recruited for the program received surveillance colonoscopy at the intervals recommended by guidelines, with annual FIT conducted between colonoscopies by individual's willing. The enrolled participants were divided into two groups: the FIT surveillance group comprised individuals who had undergone at least one FIT during the colonoscopy surveillance period, and the non‐FIT group consisted of individuals who received follow‐up colonoscopy but no FIT surveillance throughout the study period.

### 2.3 Outcomes

The primary outcomes were the incidence rates of CRC and advanced colorectal neoplasia. Advanced colorectal neoplasia was defined as CRC or advanced adenoma. Advanced adenoma was defined as at least one adenoma ≥10 mm, a villous component of at least 25%, or high‐grade dysplasia. The final clinical diagnoses and CRC stage were classified according to the American Joint Committee on Cancer (AJCC), eighth edition, stage.

### 2.4 Statistical analysis

Continuous variables are presented as mean (SD) or median (IQR), while categorical variables are reported as counts and percentages. Follow‐up time in person‐years was calculated for each group based on the date of enrollment to the date of diagnosis of outcomes or December 31, 2022, whichever occurred first. The incidence of CRC and advanced colorectal neoplasia was estimated for each group according to the number of events per 100,000 person‐years, and 95% confidence intervals (CI) were determined by the poisson distribution. To describe the observed outcomes, the unadjusted cumulative incidence was compared between groups using the Kaplan–Meier method and log‐rank tests. Cox proportional hazards regression models were used to evaluate potential associations between FIT surveillance and risk of events incidence. Hazard ratios (HRs) were reported with 95% CIs. Interaction analyses stratified by age and gender as modifiers were performed to assess potential differences in the effects of FIT across different subgroups. All statistical analyses were conducted using the R software package (version 4.1.0), with a two‐sided P value of <0.05 considered statistically significant.

### 2.5 Sensitivity analyses

Sensitivity analyses were conducted to assess/validate the effect of FIT surveillance in reducing the incidence of CRC and advanced colorectal neoplasia. First, previously studies reported that genetic factors associated with CRC could confound results, therefore participants with a family history of CRC were removed. Second, competing risk regression analyses were conducted to assess potential associations between FIT surveillance and the risk of CRC development (event) or advanced neoplasia (competing event) by the cumulative incidence function using the Fine and Gray approach, which extends the Cox model to competing risk data by considering the subdistribution hazard.

## 3 RESULTS

### 3.1 Baseline characteristics of study participants

**FIGURE 1:**
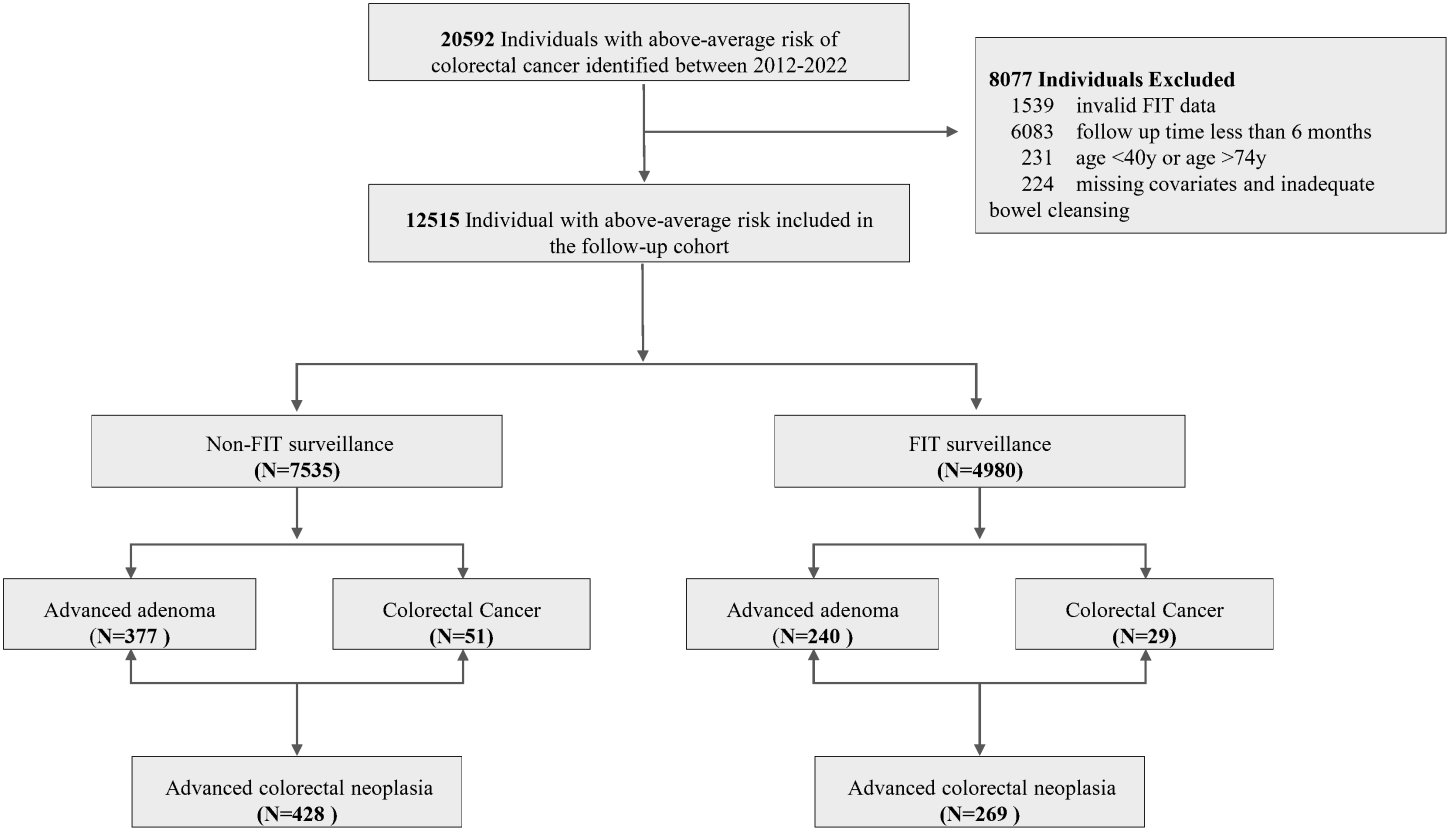
Flow diagram of the study populations. FIT, fecal immunochemical test.

The study cohort comprised a total of 12,515 individuals who had above‐average CRC risk between January 2012 and December 2022. Among them, 4980 individuals (39.79%) received at least one FIT test surveillance, the remaining 7535 individuals (60.21%) did not undergo FIT test during study period (Figure 1). More than half (53.8%) were male (6734/12515) and the mean age was 60.21 years (Table 1). The main demographic characteristics were compared between the FIT surveillance group and the non‐FIT surveillance groups. The analysis revealed no statistically significant differences between the groups in terms of family history of colorectal cancer, chronic diarrhea, chronic constipation, mucoid blood stool, chronic cholecystitis, or history of cancer.

**TABLE 1:** Baseline Characteristics of Study Participants by Surveillance Group.

Characteristic	Overall	Non‐FIT surveillance	FIT surveillance	*p* value
Total, *n*	12,515	7535	4980	
Age (mean (SD))	60.18 (7.21)	60.08 (7.32)	60.34 (7.03)	0.041
Sex (%)				
Male	6734 (53.8)	4130 (54.8)	2604 (52.3)	0.006
Female	5781 (46.2)	3405 (45.2)	2376 (47.7)	
Education (%)				
High school and below	1928 (15.4)	983 (13.0)	945 (19.0)	<0.001
High school	8461 (67.6)	5118 (67.9)	3343 (67.1)	
Postsecondary	2126 (17.0)	1434 (19.1)	692 (13.9)	
Work (%)				
Administrators and technicians	3972 (31.7)	2492 (33.1)	1480 (29.7)	<0.001
Service industry	863 (6.9)	558 (7.4)	305 (6.1)	
Production and transport devices staff	4796 (38.3)	2737 (36.3)	2059 (41.4)	
Unemployment and unknown	2884 (23.0)	1748 (23.2)	1136 (22.8)	
Previously detected colonic polyp (%)				
Yes	2679 (21.4)	1721 (22.8)	958 (19.2)	<0.001
No	9836 (78.6)	5814 (77.2)	4022 (80.8)	
Family history of colorectal cancer (%)				
Yes	1820 (14.5)	1095 (14.5)	725 (14.6)	0.988
No	10,695 (85.5)	6440 (85.5)	4255 (85.4)	
Chronic diarrhea (%)				
Yes	3292 (26.3)	1975 (26.2)	1317 (26.4)	0.786
No	9223 (73.7)	5560 (73.8)	3663 (73.6)	
Chronic constipation (%)				
Yes	3043 (24.3)	1817 (24.1)	1226 (24.6)	0.534
No	9472 (75.7)	5718 (75.9)	3754 (75.4)	
Mucoid blood stool (%)				
Yes	2721 (21.7)	1656 (22.0)	1065 (21.4)	0.445
No	9794 (78.3)	5879 (78.0)	3915 (78.6)	
Chronic appendicitis or appendectomy (%)				
Yes	1264 (10.1)	746 (9.9)	518 (10.4)	0.379
No	11,251 (89.9)	6789 (90.1)	4462 (89.6)	
Chronic cholecystitis or cholecystectomy (%)				
Yes	1257 (10.0)	755 (10.0)	502 (10.1)	0.936
No	11,258 (90.0)	6780 (90.0)	4478 (89.9)	
History of cancer (%)				
Yes	683 (5.5)	416 (5.5)	267 (5.4)	0.731
No	11,832 (94.5)	7119 (94.5)	4713 (94.6)	

Abbreviation: SD, standard deviation.

### 3.2 Association between FIT surveillance and outcomes

The total follow‐up time was 48,278 person‐years for the 12,515 participants (Table 2). The median follow‐up times of 2.9 years for CRC and 3.0 years for advanced colorectal neoplasia. During the follow‐up period, CRC was diagnosed in 80 subjects in the study population, with a cumulative incidence of 184.85 per 100,000 person‐years. Among these patients, 51 CRC cases occurred in the non‐FIT surveillance group (incidence rate, 233.88 per 100,000 person‐years), while 29 CRC cases were detected in the FIT surveillance group (incidence rate, 184.35 per 100,000 person‐years), resulting in an incidence rate ratio (IRR) of 0.58 (95% CI, 0.37–0.91). During the study period, 428 advanced colorectal neoplasia cases were reported in the non‐FIT surveillance group, while 269 cases occurred in the FIT surveillance group. A significantly lower incidence of advanced colorectal neoplasia was observed in the FIT surveillance group (IRR: 0.64; 95% CI, 0.55–0.74). The incidence rates of CRC and advanced colorectal neoplasia were higher in males and individuals older than 60 years.

**TABLE 2:** Incidence of CRC and advanced colorectal neoplasia by surveillance group.

	Total (*n* = 12,515)			Non‐FIT surveillance (*n* = 7535)			FIT surveillance (*n* = 4950)			IRR (95% CI)^a^
	Cases, no. (%)	Person years	Incidence density	Cases, no. (%)	Person years	Incidence density^b^	Cases, no. (%)	Person years	Incidence density^b^	
*Colorectal cancer*										
Total	80 (0·6%)	43,278	184.85	51 (0·7%)	21,806	233.88	29 (0·6%)	21,472	135.06	0.58 (0.37–0.91)*
Age										
40–59	8 (0·1%)	17,904	44.68	3 (5.9%)	9605	31.23	5 (17.2%)	8299	60.25	1.93 (0.46–8.07)
60–74	72 (0·5%)	25,375	283.75	48 (94.2%)	12,202	393.38	24 (82.8%)	13,173	182.19	0.46 (0.28–0.76)*
Sex										
Male	50 (0.4%)	22,759	219.69	30 (58.2%)	11,571	259.27	20 (69.0%)	11,188	178.76	0.69 (0.39–1.21)
Female	30 (0.2%)	20,519	146.20	21 (51.2%)	10,235	205.17	9 (31.0%)	10,284	87.52	0.43 (0.20–0.93)*
*Advanced colorectal neoplasia*										
Total	697 (5.6%)	43,278	1610.52	428 (5·7%)	21,806	1962.72	269 (5.4%)	21,472	1252.80	0.64 (0.55–0.74)*
Age										
40–59	205 (1.7%)	17,904	1145.02	114 (26.2%)	9605	1186.88	91 (33.8%)	8299	1096.57	0.92 (0.70–1.22)
60–74	492 (3.9%)	25,375	1938.93	314 (73.4%)	12,202	2573.35	178 (66.2%)	13,173	1351.21	0.53 (0.44–0.63)*
Sex										
Male	429 (3.4%)	22,759	1884.95	258 (60.3%)	11,571	2229.68	171 (63.6%)	11,188	1528.42	0.69 (0.57–0.83)*
Female	268 (2.2%)	20,519	1306.09	170 (39.7%)	10,235	1660.92	98 (36.4%)	10,284	952.94	0.57 (0.45–0.74)*

Abbreviations: CI, confidence interval; CRC, colorectal cancer; FIT, fecal immunochemical test; IRR, incidence rate ratio.

^a^ FIT surveillance versus non‐FIT surveillance.

^b^ Incidence density was the number of cases per 100,000 person‐years; all incidence densities are crude rates.

*Indicate statistically significant (*p* < 0.05).

**FIGURE 2:**
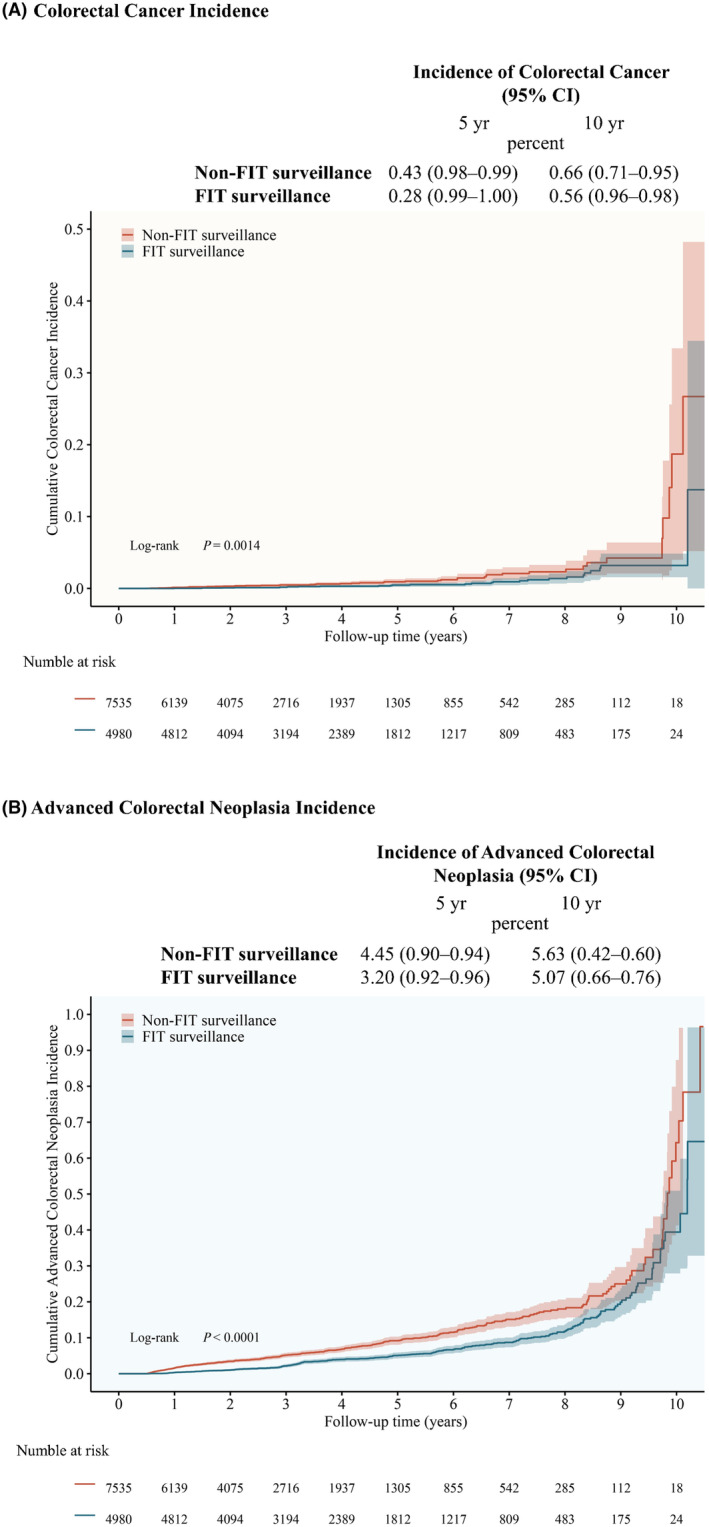
Cumulative Incidence of Colorectal Cancer and Advanced Colorectal Neoplasia by Surveillance Group. (A) Colorectal cancer. (B) Advanced colorectal neoplasia. *p* values are for the comparison between FIT surveillance and non‐FIT surveillance. FIT, fecal immunochemical test.

During the entire follow‐up, the cumulative incidence of CRC and advanced colorectal neoplasia was higher in the non‐FIT surveillance group than in the FIT group (Figure 2). After 10 years of follow‐up, the cumulative incidence of CRC was 0.66% (95% confidence interval [CI], 0.71–0.95) in the non‐FIT surveillance group and 0.56% (95% CI, 0.96–0.98) in the FIT surveillance group (Figure 2A). CRC developed in 50 participants without FIT surveillance and in 28 participants with FIT surveillance. The 10‐year cumulative incidence of advanced colorectal neoplasia was 5.63% (95% CI, 0.42–0.60) without FIT surveillance and 5.07% (95% CI, 0.66–0.76) with FIT surveillance (Figure 2B). Colorectal neoplasia developed in 424 participants without FIT surveillance and in 266 participants with FIT surveillance. Among the different groups defined by baseline colonoscopy findings, the advanced adenoma group had the highest number of cumulative events, followed by the non‐advanced adenoma group, and finally the negative colonoscopy group (Figure S2).

**FIGURE 3:**
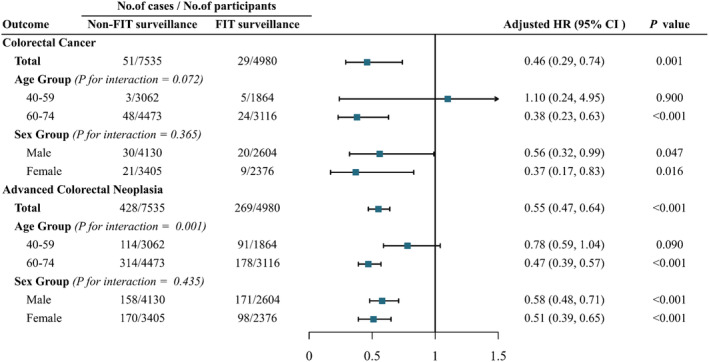
Association of FIT surveillance with colorectal cancer and advanced colorectal neoplasia stratified by age and sex. Each subgroup adjusted for multiple factors (including age, sex, education, work, a history of cancer, a history of polyps, a family history of colorectal cancer, chronic diarrhea, chronic constipation, mucoid blood stool, chronic appendicitis or appendectomy, chronic cholecystitis or cholecystectomy) except the stratification factor itself. Multiplicative interactions were assessed. FIT, fecal immunochemical test; HR, hazard ratios; CI, confidence interval.

Cox regression analyses that FIT surveillance was significantly associated with lower risk of CRC or advanced colorectal neoplasia compared with non‐FIT surveillance (Figure 3). In participants who received FIT surveillance after baseline colonoscopy, risk of CRC was significantly lower than in the non‐FIT group; risk of advanced colorectal neoplasia was similarly reduced in the FIT surveillance group. After adjusting for contributing baseline factors (age, sex, employment, education level, family history of CRC, and digestive tract symptoms), participants in the FIT surveillance group had a 54% lower risk of developing CRC (HR, 0.46; 95% CI, 0.29–0.74) and a 45% lower risk of developing advanced colorectal neoplasia (HR, 0.55; 95% CI, 0.47–0.64) compared to those in the non‐FIT surveillance group (Figure 3).

**FIGURE 4:**
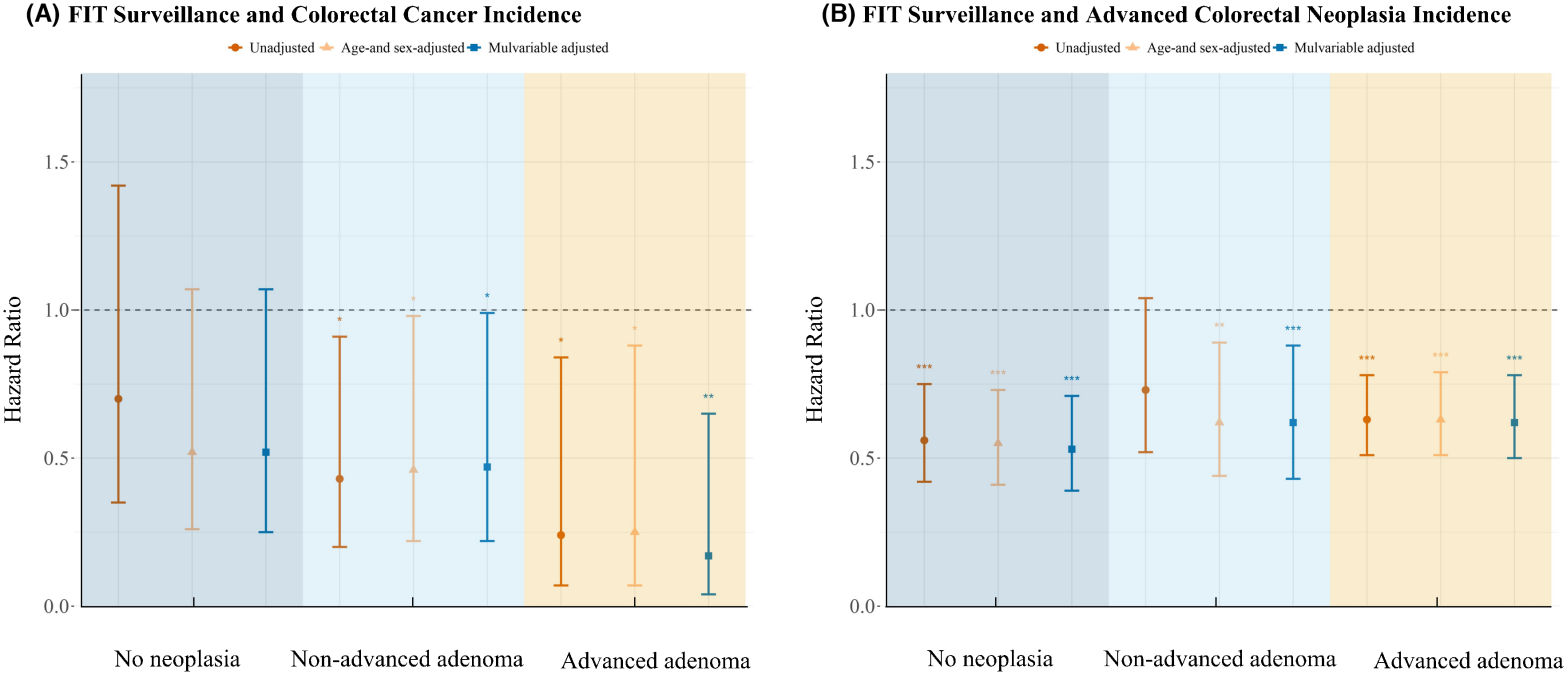
Forest plot of the adjusted hazards ratio for the association of FIT surveillance with colorectal cancer and advanced colorectal neoplasia according to prior colonoscopy findings. (A) Colorectal cancer. (B) Advanced colorectal neoplasia. Multivariable analyses were adjusted for age, sex, education, work, a history of cancer, a history of polyps, a family history of colorectal cancer, chronic diarrhea, chronic constipation, mucoid blood stool, chronic appendicitis or appendectomy, chronic cholecystitis or cholecystectomy. The statistically significance label as follows: *p < 0.05, **p < 0.01, ***p < 0.001. FIT, fecal immunochemical test; HR, hazard ratio.

Unadjusted models based on findings in the baseline colonoscopy indicated that FIT surveillance was associated with a lower risk of CRC in subjects with advanced adenoma in the initial colonoscopy (HR, 0.24; 95% CI, 0.07–0.84) (Figure 4A). Furthermore, this significant correlation was robust to adjustments for age, sex, or multivariable adjustment. In addition, the risk of CRC was also significantly reduced in individuals with non‐advanced adenoma at baseline colonoscopy. There was a reduction in the risk of colorectal cancer in individuals with no neoplasia findings in baseline colonoscopy, but it did not reach statistical significance. FIT surveillance not only reduced the occurrence of CRC but also reduced the incidence of advanced colorectal neoplasia in participants with advanced adenoma, no neoplasia, or non‐advanced adenoma at baseline (Figure 4B).

### 3.3 Subgroup analyses

FIT surveillance was also significantly associated with outcomes after stratifying for subgroups. For example, stratification by age could affect associations between FIT surveillance and advanced colorectal neoplasia (P interaction = 0.001) (Figure 3). Reduced CRC incidence (HR, 0.38; 95% CI, 0.23–0.63) and advanced colorectal neoplasia incidence (HR, 0.47; 95% CI, 0.39–0.57) was observed in FIT surveillance group participants aged 60–74 years compared with the non‐FIT surveillance group, whereas no such reduction was observed in participants aged 40–59 years. By contrast, no significant interactions were observed between FIT surveillance and sex. The incidences of both CRC and advanced colorectal neoplasia were also significantly lower in FIT surveillance groups stratified by sex than in sex‐stratified non‐FIT surveillance groups (Figure 3).

### 3.4 Sensitivity analyses

The results of sensitivity analyses were generally consistent with the above analyses, supporting that the observed effects of FIT surveillance were robust. Similar associations were found when participants with family history of CRC were excluded (Table S1). In addition, competing risk analysis, in which advanced neoplasia was considered a competing event, suggested that risk of CRC was also significantly lower in the FIT surveillance group compared to that in the non‐FIT surveillance group (HR, 0.49; 95% CI, 0.31–0.77) (Figure S2; Table S2).

## 4 DISCUSSION

In this regional retrospective cohort of participants with an above‐average risk of developing colorectal neoplasia, we observed a significant reduction in the incidence of colorectal cancer and advanced colorectal neoplasia among participants who underwent surveillance with the FIT test. These results highlight the importance of tailored surveillance approaches in reducing the burden of colorectal cancer, particularly in above‐average risk groups.

According to the current guidelines, colonoscopy is an important component of CRC surveillance,^14^, ^18^, ^19^ and post‐surgery surveillance colonoscopy has been previously associated with significantly reduced incidence of CRC.^20^ However, colonoscopy is accompanied by risks, and colonoscopy‐related complications, over‐diagnosis, and over‐treatment of CRC can result in a substantial depletion of resources for a public health system.^21^, ^22^ By contrast, FIT is a relatively simple, low‐cost tool capable of detecting fast‐growing or overlooked lesions that were missed during surveillance colonoscopy.^23^, ^24^, ^25^ Several other studies have reported evidence supporting the value of FIT test in the intervening years between surveillance colonoscopies, especially since negative FIT is also associated with lower risk for advanced neoplasia.^26^, ^27^ Despite data indicating its informative value, FIT has yet to be adopted as a routine measure in CRC surveillance because its clinical benefits in decreasing CRC incidence and potentially mortality, have remained unclear.^2^, ^28^, ^29^ Thus, the benefits of FIT surveillance are worth exploring given the potential for to improve CRC guidelines.

Our study showed that subgroups of the population who had a greater risk for advanced neoplasia in those with a diagnosis of advanced adenoma at the prime colonoscopy, those without a completed FIT in the interval, those aged over 60 years of age, and male. Previous studies have shown that individuals with above‐average risk received subsequent FIT after a negative colonoscopy can remarkably reduce the risk of incident CRC.^16^, ^17^ However, evidence based on previous colonoscopy findings related to FIT between colonoscopy interval and other important risk factors of CRC, namely age and sex remain limited. There was the highest increased risk of CRC and advanced neoplasia in those individuals who were diagnosed with advanced adenoma at the prime colonoscopy and did not receive subsequent FIT surveillance. In our study, FIT surveillance after reduced the risk of incident CRC and advanced neoplasia stratifying participants according to prime colonoscopy findings. Overall, we found that those with advanced adenoma were more likely to highly benefit from FIT surveillance.

FITs present important value in colonoscopy surveillance, and might have identified the missed lesion to prevent colorectal cancer When an advanced adenoma was previously missed by colonoscopy,^30^, ^31^ especially in some critical settings when resources are constrained by healthcare funding or events, such as the pandemic, implementing FIT to extend colonoscopy intervals would help relieve and lessen the backlog of colonoscopy and distribute health resources for ensuring rational.^32^ Therefore, based on the results of our present study, we propose that above‐average risk individuals after colonoscopy should routinely receive a subsequent FIT to detect missed cancers and advanced adenomas.

This study has several strengths and limitations. First, it was based on a large sample size, community‐based recruitment; validated approaches to capture pathology data and colonoscopy examinations; adjustment of major factors that might have affected the risk for incident CRC to make precise estimations. Second, these enrolled subjects underwent FIT between the repeat colonoscopies. The conclusion presented in this study may be applicable to similar situations for colonoscopy surveillance programs in different areas, including individuals with a family history of colorectal cancer, personal history of colorectal adenomas, or positive fecal occult blood test as well. Third, the sufficient follow‐up time of the cohort is taken into consideration, which is needed for the occurrence and progression of outcome events and the sojourn time of preclinical colorectal cancer. In addition, our study included diverse evaluation of assumptions through sensitivity analyses. Our study also has some limitations. First, although we found that FIT surveillance after colonoscopy is associated with the risk of CRC, the result cannot prove causality arises from limitations of any observational study. Second, one limitation of our study is self‐selection bias. Besides, some important confounders such as adenoma detection rate and lifestyle factors such as smoking or physical activity have not been measured and adjusted in this retrospective cohort study. Therefore, the residual confounding by measured factors may still be present. Third, the effect of more than one subsequent FIT on incident CRC has yet to be explored. Going forward, we will persist the surveillance cohort and keep providing up‐to‐date and vital information, including tumor stages and treatments and survival rates based on longer follow‐up periods, controlling for more objective measurements and adjusting covariates. Further, we will explore to clarify the appropriate timing and frequency of FIT follow‐up and identify the population that may benefit from this approach.

## 5 CONCLUSION

This cohort study supports FIT as surveillance tool for above‐average risk individuals, as they have a higher risk of developing colorectal neoplasia than the general population. Additionally, FIT surveillance is associated with a reduction in CRC and advanced colorectal neoplasia incidence. Our study supporting the potential of FIT surveillance as an effective strategy for reducing the incidence of colorectal cancer and advanced colorectal neoplasia in above‐average risk population.

## AUTHOR CONTRIBUTIONS

**Hai Qin:** Data curation (equal); formal analysis (equal); writing – original draft (equal); writing – review and editing (equal). **Mingqing Zhang:** Data curation (supporting); formal analysis (equal); writing – original draft (equal); writing – review and editing (equal). **Guanglu Zhang:** Formal analysis (equal); visualization (equal); writing – original draft (equal); writing – review and editing (equal). **Lizhong Zhao:** Data curation (supporting); investigation (equal); validation (equal). **Huan Zhang:** Data curation (supporting); investigation (supporting); validation (equal). **Weituo Zhang:** Validation (equal); writing – review and editing (supporting). **Yijia Wang:** Formal analysis (supporting); writing – review and editing (supporting). **Xipeng Zhang:** Conceptualization (equal); methodology (equal); writing – review and editing (supporting). **Li Xie:** Formal analysis (equal); methodology (equal); writing – original draft (equal); writing – review and editing (equal). **Biyun Qian:** Conceptualization (lead); funding acquisition (lead); methodology (lead); project administration (lead); resources (lead); supervision (lead); writing – review and editing (lead).

## CONFLICT OF INTEREST STATEMENT

The authors declare that they have no conflicts of interest.

## Supporting information


Data S1.

## Data Availability

The datasets generated and/or analyzed during the current study are not publicly available due to privacy and ethical restrictions but are available from the corresponding author on reasonable request.
