# Supplementary material for: Enhancing colorectal cancer screening in high‐risk population through fecal immunochemical test surveillance: Results from a surveillance program
Source: Cancer Med. 2024 Oct 20;13(20):e70145. doi: 10.1002/cam4.70145 (PMC11491543; doi:10.1002/cam4.70145)
Supplement: Supplementary file 1 — Data S1. [file CAM4-13-e70145-s001.docx]

**Supplementary Content**

**eFigure 1. Cumulative incidence according to prior colonoscopy findings. (A) colorectal cancer. (B) advanced colorectal neoplasia.**

**eFigure 2. Cumulative incidence colorectal cancer using competing risk analysis. Colorectal cancer as an interest event and advanced neoplasia as a competing event.**

**eTable 1. Association between FIT surveillance and outcomes incidence excluding participants with a family history of colorectal cancer (N=10695).**

**eTable 2. Association between FIT surveillance and Colorectal Cancer risk using competing risk analysis considering advanced neoplasia as a competing event.**

**eFigure 1. Cumulative incidence according to prior colonoscopy findings.** (A) colorectal cancer. (B) advanced colorectal neoplasia.

Abbreviations: FIT, faecal immunochemical test.


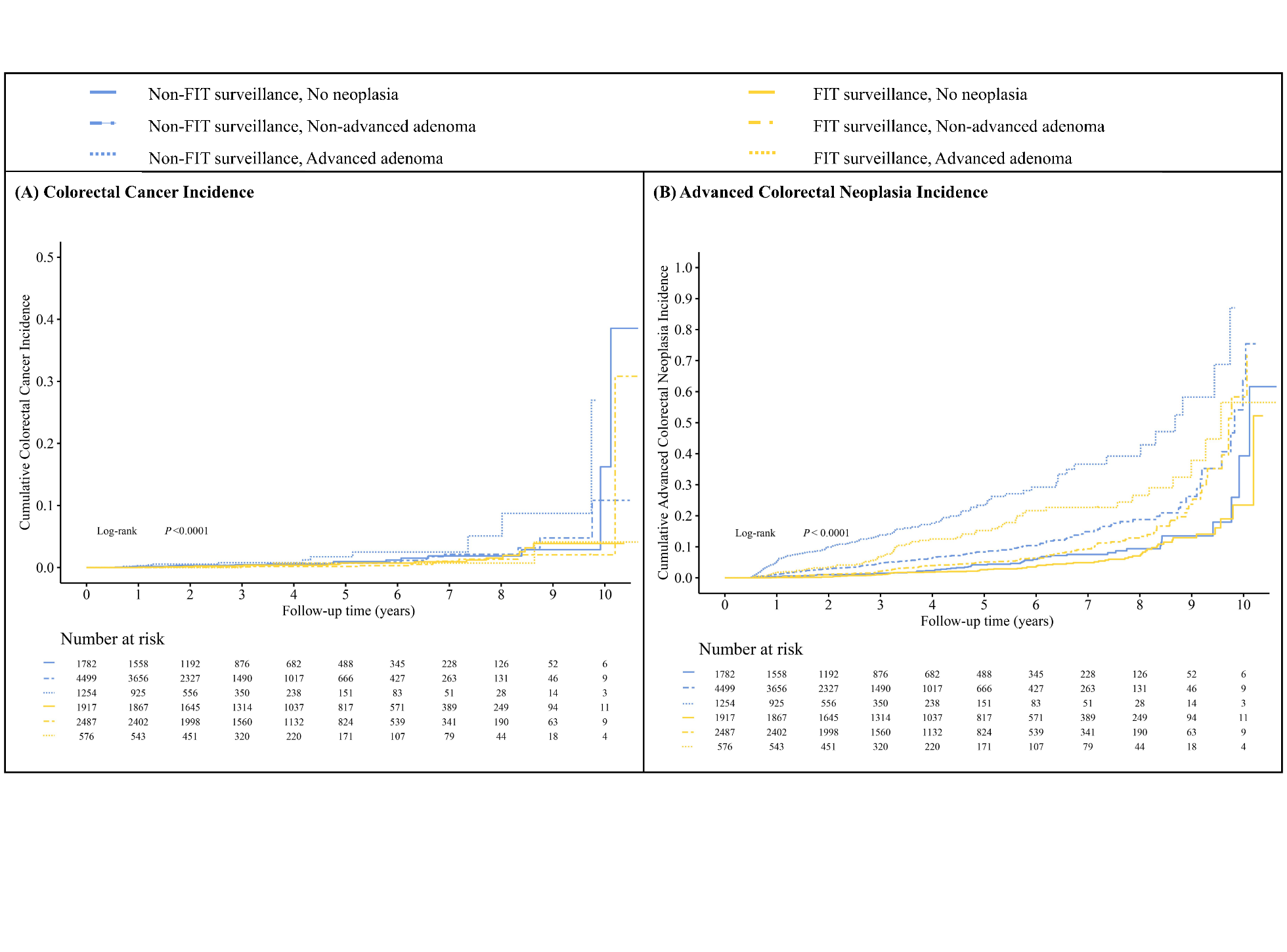


**eFigure 2. Cumulative incidence colorectal cancer using competing risk analysis.**

Colorectal cancer as an interest event and advanced neoplasia as a competing event.

Note: Main analysis: enlarged view; Competing risk analysis: thumbnail image. Abbreviations: FIT, faecal immunochemical test.


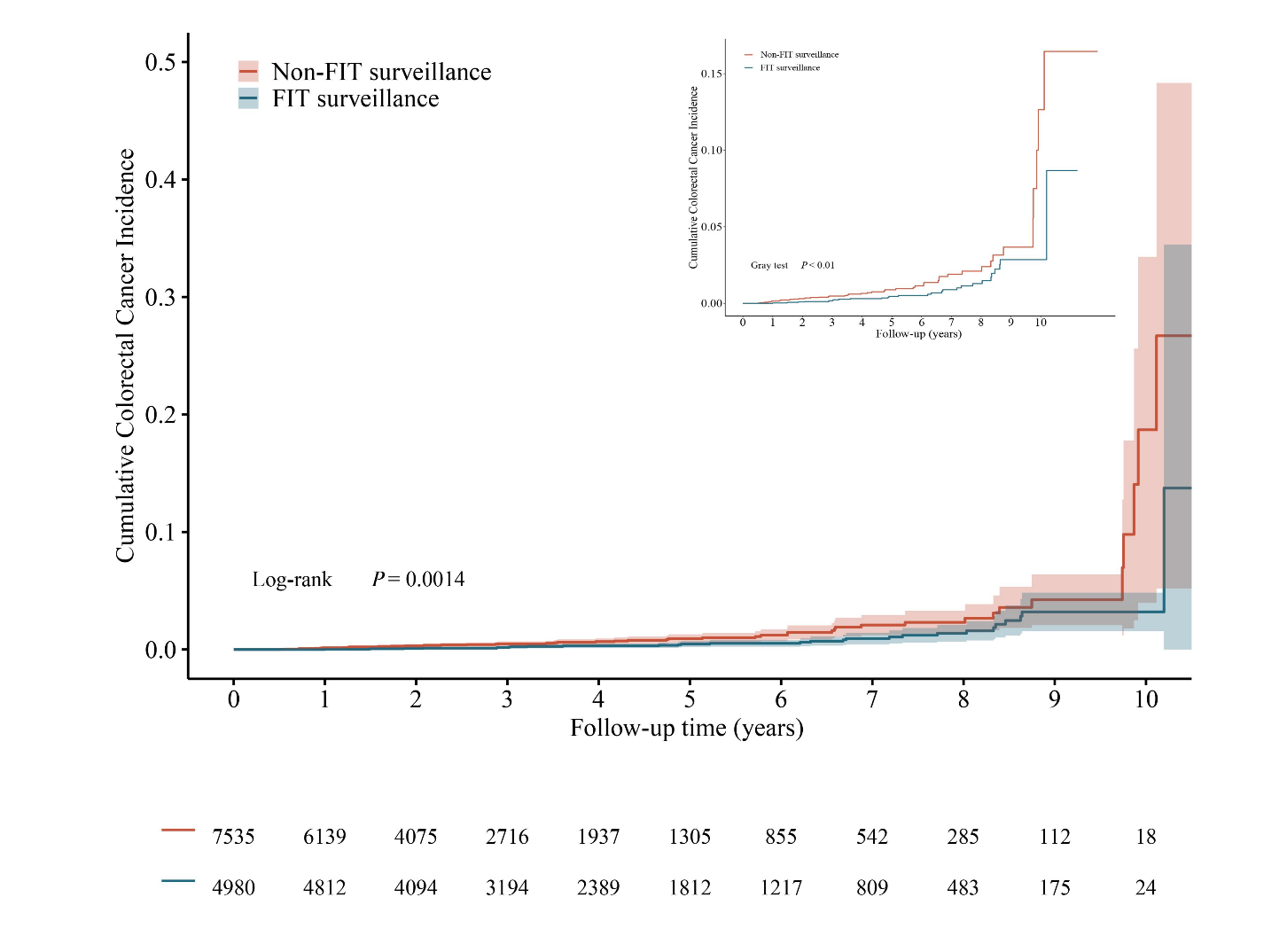


**eTable 1. Association between FIT surveillance and outcomes incidence excluding participants with a family history of colorectal cancer (N=10695).**

| **Outcome** | **No.of Cases / No.of Participants** | **Adjusted HR (95% CI )** | ***P* value** |
| --- | --- | --- | --- |
| ***Colorectal Cancer*** | | | |
| **Surveillance** |  |  |  |
| Non-FIT surveillance | 46/6440 | 1.00 (Reference) | 1.00 (Reference) |
| FIT surveillance | 27/4255 | 0.49(0.30, 0.79) | 0.003 |
| **Age Group** |  |  |  |
| 40-59 group |  |  |  |
| Non-FIT surveillance | 1/2594 | 1.00 (Reference) | 1.00 (Reference) |
| FIT surveillance | 5/1587 | 3.06(0.34, 27.2) | 0.315 |
| 60-74 group |  |  |  |
| Non-FIT surveillance | 45/3846 | 1.00 (Reference) | 1.00 (Reference) |
| FIT surveillance | 22/2668 | 0.37(0.22, 0.63) | <0.001 |
| **Sex Group** |  |  |  |
| Male group |  |  |  |
| Non-FIT surveillance | 26/3579 | 1.00 (Reference) | 1.00 (Reference) |
| FIT surveillance | 18/2244 | 0.59(0.32, 1.08) | 0.088 |
| Female group |  |  |  |
| Non-FIT surveillance | 20/2861 | 1.00 (Reference) | 1.00 (Reference) |
| FIT surveillance | 9/2011 | 0.39(0.18, 0.88) | 0.023 |
| ***Advanced Colorectal Neoplasia*** | | | |
| **Surveillance** |  |  |  |
| Non-FIT surveillance | 367/6440 | 1.00 (Reference) | 1.00 (Reference) |
| FIT surveillance | 236/4255 | 0.57(0.48, 0.67) | <0.001 |
| **Age Group** |  |  |  |
| 40-59 group |  |  |  |
| Non-FIT surveillance | 93/2594 | 1.00 (Reference) | 1.00 (Reference) |
| FIT surveillance | 80/1587 | 0.86(0.63, 1.16) | 0.324 |
| 60-74 group |  |  |  |
| Non-FIT surveillance | 274/3846 | 1.00 (Reference) | 1.00 (Reference) |
| FIT surveillance | 156/2668 | 0.48(0.40, 0.59) | <0.001 |
| **Sex Group** |  |  |  |
| Male group |  |  |  |
| Non-FIT surveillance | 220/3579 | 1.00 (Reference) | 1.00 (Reference) |
| FIT surveillance | 150/2244 | 0.61(0.50, 0.76) | <0.001 |
| Female group |  |  |  |
| Non-FIT surveillance | 147/2861 | 1.00 (Reference) | 1.00 (Reference) |
| FIT surveillance | 86/2011 | 0.53(0.40, 0.69) | <0.001 |

Analyses were adjusted for age, sex, education, work, a history of cancer, a history of polyps, a family history of colorectal cancer, chronic diarrhea, chronic constipation, mucoid blood stool, chronic appendicitis or appendectomy, chronic cholecystitis or cholecystectomy.

Abbreviations: FIT, faecal immunochemical test; HR, hazard ratio; CI, confidence interval

**eTable 2. Association between FIT surveillance and Colorectal Cancer risk using competing risk analysis considering advanced neoplasia as a competing event.**

| **Subgroup** | **No.of Cases / No.of Participants** | **Unadjusted HR (95% CI )** | **Adjusted HR (95% CI )** |
| --- | --- | --- | --- |
| **Surveillance** |  |  |  |
| Non-FIT surveillance | 51/7535 | 1.00 (Reference) | 1.00 (Reference) |
| FIT surveillance | 29/4980 | 0.50(0.32, 0.78) | 0.49(0.31, 0.77) |
| **Age Group** |  |  |  |
| 40-59 group | 3/3062 | 1.00 (Reference) | 1.00 (Reference) |
| Non-FIT surveillance | 5/1864 | 1.52(0.39, 5.94) | 1.16(0.27, 4.92) |
| FIT surveillance |  |  |  |
| 60-74 group |  |  |  |
| Non-FIT surveillance | 48/4473 | 1.00 (Reference) | 1.00 (Reference) |
| FIT surveillance | 24/3116 | 0.41(0.25, 0.66) | 0.40(0.25, 0.66) |
| **Sex Group** |  |  |  |
| Male group |  |  |  |
| Non-FIT surveillance | 30/4130 | 1.00 (Reference) | 1.00 (Reference) |
| FIT surveillance | 20/2604 | 0.60(0.35, 1.06) | 0.59(0.34, 1.03) |
| Female group |  |  |  |
| Non-FIT surveillance | 21/3405 | 1.00 (Reference) | 1.00 (Reference) |
| FIT surveillance | 9/2376 | 0.37(0.17, 0.79) | 0.39(0.18, 0.84) |

Analyses were adjusted for age, sex, education, work, a history of cancer, a history of polyps, a family history of colorectal cancer, chronic diarrhea, chronic constipation, mucoid blood stool, chronic appendicitis or appendectomy, chronic cholecystitis or cholecystectomy.

Abbreviations: FIT, faecal immunochemical test; HR, hazard ratio; CI, confidence interval.
